# A Novel Swarm Intelligence—Harris Hawks Optimization for Spatial Assessment of Landslide Susceptibility

**DOI:** 10.3390/s19163590

**Published:** 2019-08-17

**Authors:** Dieu Tien Bui, Hossein Moayedi, Bahareh Kalantar, Abdolreza Osouli, Biswajeet Pradhan, Hoang Nguyen, Ahmad Safuan A Rashid

**Affiliations:** 1 Institute of Research and Development, Duy Tan University, Da Nang, Vietnam; 2 Department for Management of Science and Technology Development, Ton Duc Thang University, Ho Chi Minh City, Vietnam; 3 Faculty of Civil Engineering, Ton Duc Thang University, Ho Chi Minh City, Vietnam; 4 RIKEN Center for Advanced Intelligence Project, Goal-Oriented Technology Research Group, Disaster Resilience Science Team, Tokyo 103-0027, Japan; 5 Civil Engineering Department, Southern Illinois University, Edwardsville, IL 62026, USA; 6 Centre for Advanced Modelling and Geospatial Information Systems (CAMGIS), Faculty of Engineering and IT, University of Technology Sydney, Ultimo, NSW 2007, Australia; 7 Department of Energy and Mineral Resources Engineering, Choongmu-gwan, Sejong University, 209 Neungdong-ro, Gwangjin-gu, Seoul 05006, Korea; 8 Department of Surface Mining, Hanoi University of Mining land Geology, 18 Vien Street, Duc Thang Ward, Bac Tu Liem District, Hanoi, Vietnam; 9 Center for Mining, Electro-Mechanical Research, Hanoi University of Mining and Geology, 18 Vien Street, Duc Thang Ward, Bac Tu Liem District, Hanoi, Vietnam; 10 Centre of Tropical Geoengineering (Geotropik), School of Civil Engineering, Faculty of Engineering, Universiti Teknologi Malaysia, Johor Bahru 81310, Malaysia

**Keywords:** landslide susceptibility mapping, GIS, artificial neural network, Harris hawks optimization

## Abstract

In this research, the novel metaheuristic algorithm Harris hawks optimization (HHO) is applied to landslide susceptibility analysis in Western Iran. To this end, the HHO is synthesized with an artificial neural network (ANN) to optimize its performance. A spatial database comprising 208 historical landslides, as well as 14 landslide conditioning factors—elevation, slope aspect, plan curvature, profile curvature, soil type, lithology, distance to the river, distance to the road, distance to the fault, land cover, slope degree, stream power index (SPI), topographic wetness index (TWI), and rainfall—is prepared to develop the ANN and HHO–ANN predictive tools. Mean square error and mean absolute error criteria are defined to measure the performance error of the models, and area under the receiving operating characteristic curve (AUROC) is used to evaluate the accuracy of the generated susceptibility maps. The findings showed that the HHO algorithm effectively improved the performance of ANN in both recognizing (AUROC_ANN_ = 0.731 and AUROC_HHO–ANN_ = 0.777) and predicting (AUROC_ANN_ = 0.720 and AUROC_HHO–ANN_ = 0.773) the landslide pattern.

## 1. Introduction

Landslides are defined as gravity-triggered downward mass movements which can result from anthropogenic and natural activities [1,2]. They are considered as one of the most devastating environmental threats and have cause huge physical and financial damage worldwide [3]. In Iran, landslide hazard is responsible for the loss of around 187 lives [4]. Due to geographical conditions, the west of Iran is known as a landslide-prone area. In the last few decades, the Seimareh landslide which occurred in 1998, has recorded the largest debris flow [5]. Hence, being able to reliably predict the landslide susceptibility of such areas can be effective in dealing with this natural hazard [6]. 

Landslide susceptibility maps the spatial occurrence likelihood of the landslide when a number of geoenvironmental factors are involved [7]. During recent years, various landslide predictive models have been developed to explore the relationship between the landslide and its conditioning (geological, topographical, and other geoenvironmental) factors. Generally, these methods can be grouped into three major classes, namely, physical strategies, expert system, and data mining techniques [8]. Among those, the third group approaches, which are based on statistical methods and machine learning strategies, are known as the most efficient landslide predictive models [9]. Many studies have produced the landslide susceptibility map of different study areas using statistical and multicriteria decision methods [10,11,12,13]. More recently, capable intelligent tools like artificial neural network (ANN), support vector machine (SVM), and adaptive neuro-fuzzy inference system (ANFIS) have been successfully used for modeling the landslide susceptibility [14,15,16,17,18,19,20]. Pham et al. [21] assessed the efficiency of three machine learning tools, namely functional tree (FT), multilayer perceptron (MLP), and naïve Bayes (NB) for producing the landslide susceptibility map of the Uttarakhand Area, India. According to their findings, the MLP (85% accuracy) presented the most accurate prediction of landslide compared to FT (84.9% accuracy) and NB (83.8% accuracy. Moreover, in a comparative study, Bui et al. [22] used SVM, MLP, radial basis function neural networks (RBF), logistic model trees (LMT), and kernel logistic regression (KLR) for analyzing the landslide susceptibility in Son La hydropower basin of Vietnam. They showed that the MLP (90.2% accuracy) outperformed SVM, KLR, RBF, and LMT with 88.7%, 87.9%, 87.1%, and 86.1% accuracy, respectively. 

Moreover, many scholars have used various hybrid metaheuristic algorithms for the prevailing computational drawbacks (e.g., local minimum and dimension dangers [23]) of the mentioned intelligent models [24,25,26,27,28,29,30,31]. In this regard, Moayedi et al. [32] applied particle swarm optimization (PSO) to an MLP neural network for landslide susceptibility modeling in the Kermanshah Province, Iran. It was shown that the PSO decreases the performance error of the MLP and the PSO–ANN achieves a more accurate susceptibility map. Likewise, Nguyen et al. [33] showed the applicability of artificial bee colony (ABC) and PSO metaheuristic techniques in optimizing the ANN for landslide susceptibility mapping in Golestan Province, Iran. Regarding the obtained area under the curves (AUCs) of 76.60%, 85.70%, and 80.30%, respectively for the ANN, PSO–ANN, and ABC–ANN, they concluded that the mentioned algorithms are capable enough to improve the ANN.

This study employs a novel hybrid optimization technique, namely Harris hawks optimization (HHO) algorithm, for finding the most appropriate computational parameters of the ANN for susceptibility assessment of a landslide-prone area in the west of Iran. This technique has not been previously used for this purpose. In this sense, the accuracy of the landslide susceptibility maps produced by the optimized and non-optimized ANNs are compared in various ways to evaluate the effect of the HHO algorithm.

## 2. Study Area

**Figure 1:**
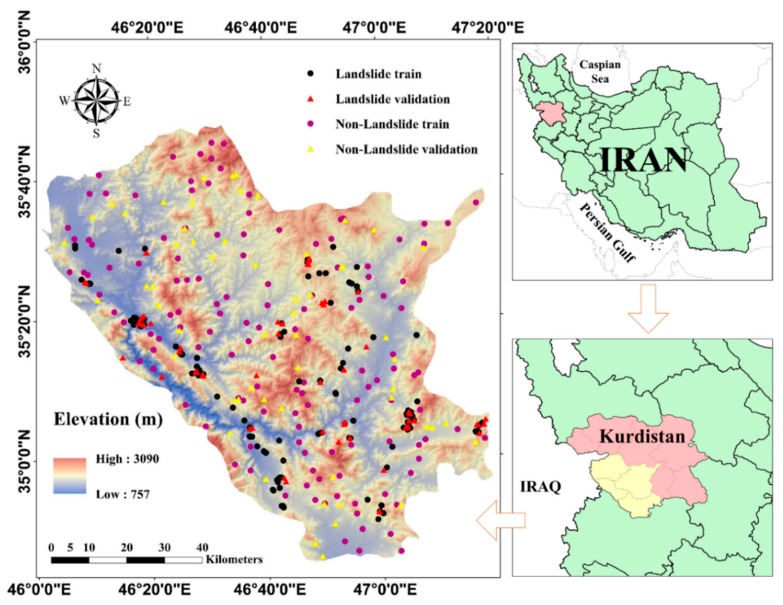
Location of the study area and distribution of the landslides.

The study area is located in the southern part of the Kurdistan Province, west of Iran (Figure 1). Geographically, it lies between the longitude 46°00’ to 47°20’ E, and the latitude 34°45’ to 35°48’ N, covering roughly 7811 km^2^. It also contains the three cities Marivan, Sanandaj, and Kamyaran. Due to the proximity to the Zagros Mountains, Kurdistan is a mountainous region. Two distinct climates can be found in this area, including semi-arid with cold winters and temperate climate, affecting the mountainous regions and high plains, respectively [34]. Dry farming and irrigated agriculture play a significant role in the economy of the people living in this area [35]. Moreover, the altitude varies approximately from 750 to 3100, where more than 80% of the area is above 1500 m.s.l. The terrain slope ranges from 0 to 60°, where more than 55% are classified as gentle slopes (i.e., lower than 15°). Also, the land is mainly covered by good ranges. Geologically, among the diverse kinds of lithological units, the so-called category “dark grey argillaceous shale” is the most common representing around 18% of the bedrock area. 

## 3. Data Preparation and Spatial Relationship between the Landslide and Related Factors

As is known, providing a valid dataset is a crucial step of using artificial intelligence tools [9]. The spatial database of this research comprises a landslide inventory map as the target, and fourteen landslide conditioning factors as the input variables, namely elevation, slope aspect, plan curvature, profile curvature, soil type, lithology, distance to the river, distance to the road, distance to the fault, land cover, slope degree, stream power index (SPI), topographic wetness index (TWI), and rainfall. Notably, plan curvature and profile curvature are topographical attributes which contribute to the convergence (or divergence) of the flowing water and the velocity change of the flowing mass, respectively [36,37,38]. A total of 208 historical landslides were identified, and the same number of the non-landslide points were randomly produced over the study area. 

**Figure 2:**
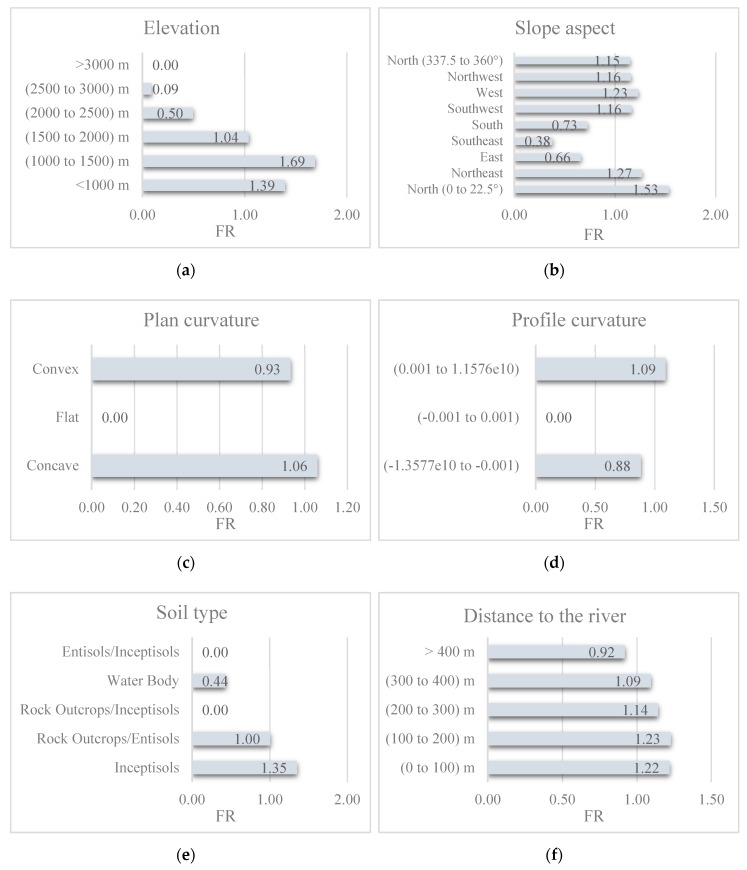

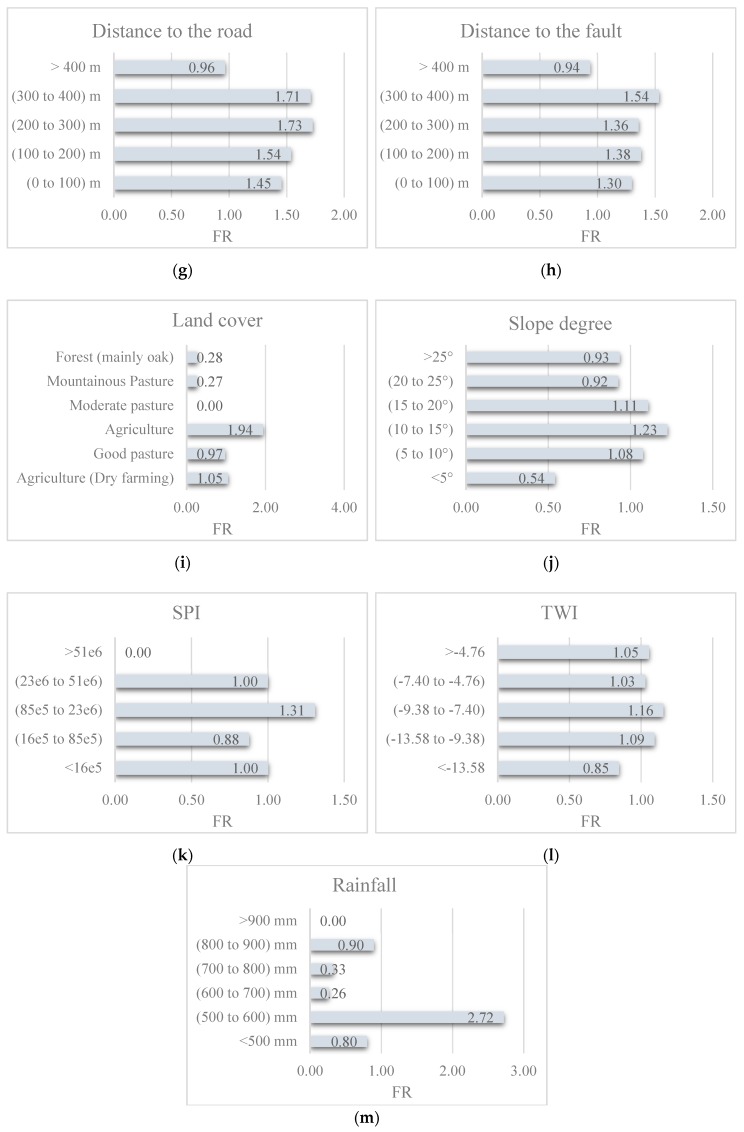
The calculated frequency ratio(FR) for (**a**) elevation, (**b**) slope aspect, (**c**) plan curvature, (**d**) profile curvature, (**e**) soil type, (**f**) distance to river, (**g**) distance to road, (**h**) distance to fault, (**i**) land cover, (**j**) slope degree, (**k**) SPI, (**l**) TWI, and (**m**) rainfall.

All layers were created from basic sources (e.g., satellite imagery and vector data) and processed in geographic information system (GIS) with a pixel size of 10 m × 10 m [34,39]. In the following, frequency ratio (FR) theory is used to evaluate the spatial relationship between the landslide and each subclass of the conditioning factors. In this sense, the higher values indicate a greater correlation between the landslide and the proposed subclass [40]. Equation (1) expresses the FR formulation:

(1) $$FR=\;\frac{N_{landslide}}{N_{domain}},$$

where *N_landslide_* and *N_domain_* respectively stand for the percentage of the landslides found in the proposed subclass and the percentage of the terrain it covers. Figure 2 illustrates the classification of the considered conditioning factors as well as the calculated FR. Also, the description of the geological units is presented in Table 1. As is seen, the largest FRs are obtained for (a) 1000–1500 m for elevation, (b) North (0–22.5°) for slope aspect, (c) “Concave” for plan curvature, (d) (0.001 − 1.1576 × 10^10^) for profile curvature, (e) “Inceptisols” for soil type, (f) 100–200 m for distance to the river, (g) 200–300 m for distance to the road, (h) 300–400 m for distance to the fault, (i) “Agriculture” for land cover, (j) 10–15° for slope, (k) 85 × 10^5^–23 × 10^6^ for SPI, (l) −9.38 to −7.40 for TWI, (m) 500–600 mm for rainfall, and (n) “Jugr” for lithology. Note that explaining the pattern of the obtained FRs requires exact analysis of the conditioning factors, like importance evaluation, which is not the aim of this paper.

## 4. Methodology

**Figure 3:**
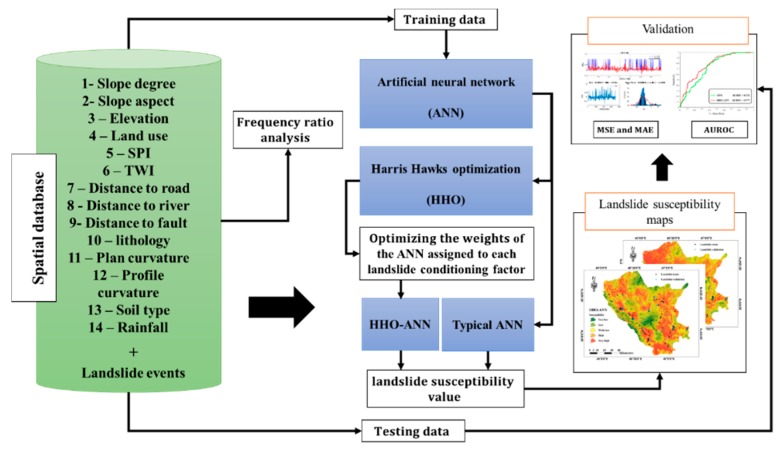
Applied procedure for landslide susceptibility assessment of this study.

The overall steps carried out in this research are shown in Figure 3. After providing the required landslide inventory map, as well as landslide conditioning layers, the marked landslides are randomly divided into two separate parts, including 70% of whole data (146 landslides and 146 non-landslide points) for training the ANN and HHO–ANN models, and 30% of data (62 landslides and 62 non-landslide points) for evaluating their efficiency in predicting future landslides in the programming language of MATLAB 2014. The HHO is then coupled with ANN to optimize its computational parameters. The landslide susceptibility maps are produced, and the accuracy of each model is evaluated by three criteria, namely area under the receiving operating characteristic curve (AUROC), mean square error (MSE), and mean absolute error (MAE). Equations (2) and (3). denote the MSE and MAE in which *N* shows the number of involved samples, and *Y_i_observed__* and *Y_i_predicted__* are the desired and predicted values of landslide susceptibility, respectively.

(2) $$MSE=\;\frac{1}{N}\overset{N}{\underset{i=1}{\sum }}{\left(Y_{i_{observed}\;}-Y_{i_{predicted}\;}\right)}^{2}$$

(3) $$MAE=\;\frac{1}{N}\overset{N}{\underset{i=1}{\sum }}\left|Y_{i_{observed}\;}-Y_{i_{predicted}\;}\right|$$

### 4.1. Artificial Neural Network

**Figure 4:**
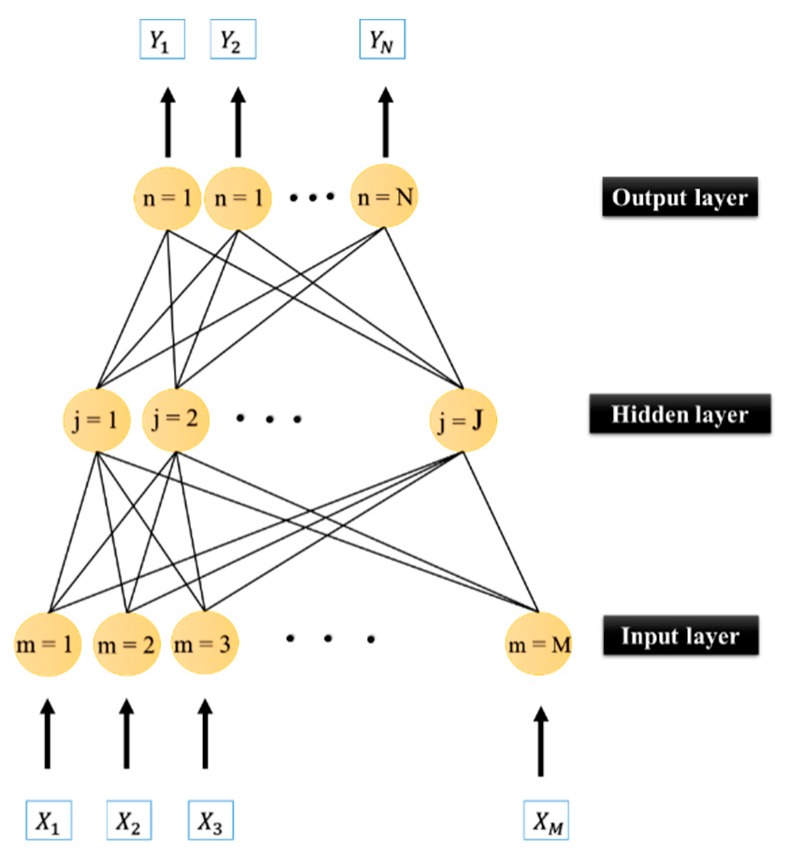
The general structure of the ANN.

Inspired by the biological interaction in the neural systems, artificial neural network (ANN) was first suggested by McCulloch and Pitts [41] as a predictive tool. It is constructed from some extremely connected units which aim to discern the nonlinear relationship between external inputs [42]. The main reason for selecting the ANN as the basic model of this study was its high capability in various engineering simulations [43,44,45,46,47]. Figure 4 illustrates the structure of a commonly held type of neural networks named multilayer perceptron (MLP). The learning process is carried out by assigning some weights to each input vector. After producing the network response, the determined weights and biases are adjusted to minimize the error. More specifically, when the output ($O_{z})$ is produced, $\chi_{z}$ is computed as follows:

(4) $$\chi_{z}=O_{z}(1-O_{z})\;(O_{z}-\;T_{z}),$$

where $T_{z}$ is the desired response for the output node *z*.

Then, the parameter $\lambda_{s}$ is calculated for each node of the middle layer as follows:

(5) $$\lambda_{s}=O_{s}(1-O_{s})\;\sum_{z}\chi_{z}W_{sz},$$

in which $W_{sz}$ represents the connecting weight between the neurons *s* and *z*.

Next, considering *m* as the learning rate (ranging from 0 to 1), two parameters below are calculated to change the weights and biases in layer *L*:

(6) $$\Delta W=-\;m\;\chi_{L}\;L_{L-1},$$

(7) $$\Delta b=-\;m\;\chi_{L}.$$

Eventually, the new weights and biases are produced as follows:

(8) $$W_{new}\;=\;W\;+\;\Delta W,$$

(9) $$b_{new}\;=\;b\;+\;\Delta b.$$

### 4.2. Harris Hawks Optimization

**Figure 5:**
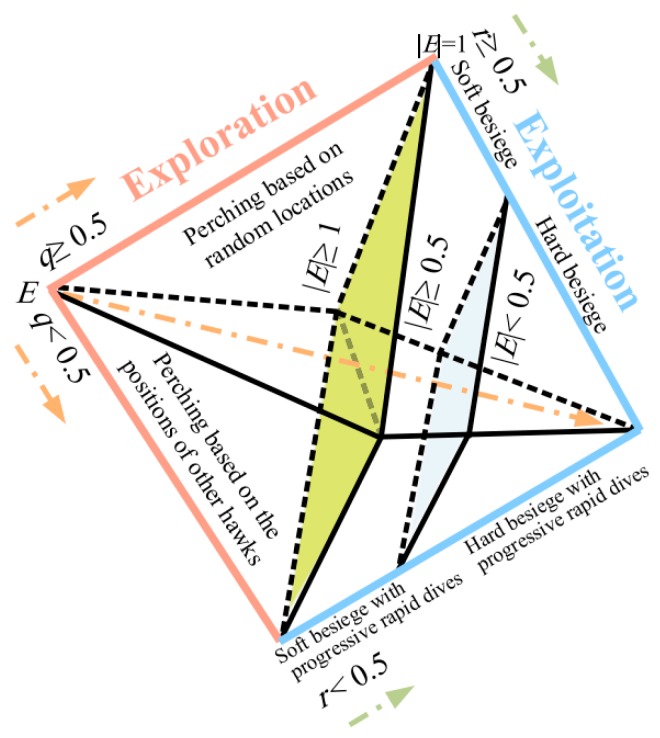
Different phases of Harris hawks optimization (after Heidari et al. [48]).

Inspired by the chasing style and cooperative behavior of Harris’ hawks, the Harris hawks optimization (HHO) technique was suggested by Heidari et al. [48]. Some of the hawks aim to surprise the prey by swooping it from different paths. Note that they select the chase pattern based on the flying pattern of the prey. HHO is a population-based search algorithm which draws on three major steps which are explained as follows (see Figure 5):

(i) Exploration Phase

In this phase, it is determined to mathematically wait, search, and discover the desired hunt. The *iter + 1* (the Harris hawks position) is mathematically expressed as follows:

(10) $$\begin{array}{l} X\left(iter+1\right)\\ =\{\begin{array}{ll} X_{rand}\left(iter\right)-r_{1}|X_{rand}\left(iter\right)-2r_{2}X\left(iter\right) & if\;q\geq 0.5\\ \left(X_{rabit}\left(iter\right)-X_{m}\left(iter\right)\right)-r_{3}\left(LB+r_{4}\left(UB-LB\right)\right) & if\;q<0.5 \end{array} \end{array}$$

where $X_{rabit}$ stands for the rabbit position, *iter* denotes the present iteration, $X_{rand}$ is the randomly selected hawk among the available population, $r_{i}$, I = 1, 2, 3, 4, q are random numbers ranging in [0, 1], and $X_{m}$ shows the average position for hawks and is computed as follows:

(11) $$X_{m}\left(iter\right)=\frac{1}{N}\overset{N}{\underset{i=1}{\sum }}X_{i}\left(iter\right),$$

in which $X_{i}$ shows the place of the hawks and N represents the hawk size.

(ii) Transition from Exploration to Exploitation

Considering *T* as the maximum size about the repetitions and $E_{0}\in \left(-1,\;1\right)$ as the initial energy during each step, HHO calculates the escaping energy of rabbit (*E*) by Equation (12). Regarding this value, exploration and exploitation may be changed.

(12) $$E=2E_{0}\left(1-\frac{iter}{T}\right)$$

In this sense, if |E|≥1, the exploration phase gets started; otherwise, the neighborhood of the solutions is aimed to be exploited.

(iii) Exploitation Phase

Depending on the residual energy of the prey, the hawks may consider a soft or hard besiege for hunting it from different directions. A so-called parameter “*r*” is defined to measure the escaping chance of the prey. Accordingly, r<0.5 represents a successful escape. In addition, when |E|≥0.5, HHO takes soft surround and when |E|<0.5, hard surround is applied. It is worth noting that even if the prey is able to escape (i.e., |E|≥0.5), its success also depends on *r*. The attack procedure is influenced by the escaping and pursuing strategy of the prey and hawks, respectively. In this sense, four major steps are considered which are broadly explained in the Appendix A [49].

## 5. Results and Discussion

### 5.1. Model Implementation

**Figure 6:**
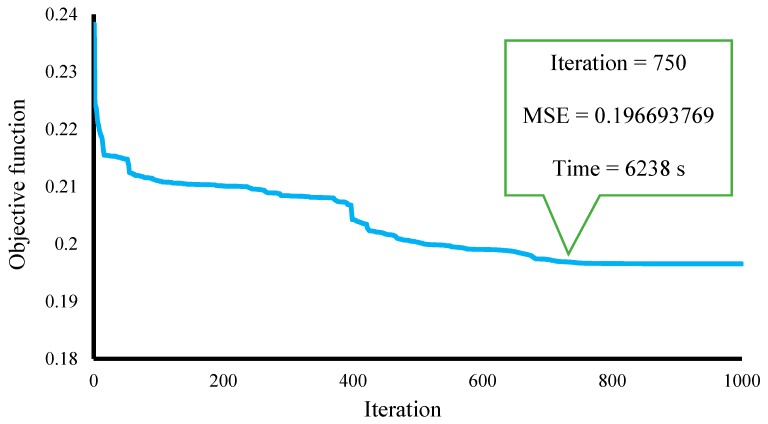
The convergence cure of the applied HHO–ANN model.

As stated above, this study addresses novel optimization of the artificial neural network based on the Harris hawks optimization technique for spatial prediction of a landslide. To this end, the HHO is synthesized with a multilayer perceptron neural network for finding the most appropriate computational parameters. More specifically, the ANN calculates the outputs by assigning some weights and biases. For optimization purposes, the structure of the ANN is mathematically introduced to the evolutionary algorithms. Here, the proposed HHO aims to find a solution containing the best alternatives to the weights and biases of the unreinforced ANN. It is worth noting that based on the authors’ experience as well as a trial and error process, the proposed ANN was constructed with five hidden neurons. Also, “Tansig” was considered as their activation function. The proposed HHO–ANN ensemble was performed within 1000 repetitions when the MSE was defined as the objective function (OF). The model executed all 1000 iterations within 8370 s and reduced the OF from 0.238274922 to 0.196520013. However, the majority was decreased before the 750th try. Figure 6 shows the convergence curve of the proposed HHO–ANN.

### 5.2. Landslide Susceptibility Mapping

**Figure 7:**
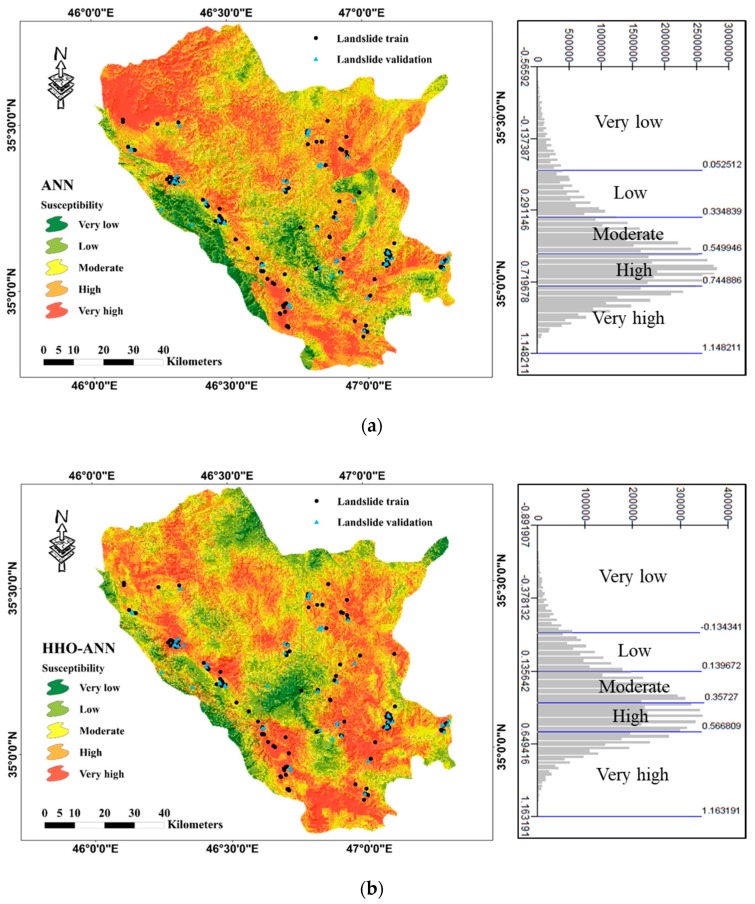
Landslide susceptibility maps developed by (**a**) ANN and (**b**) HHO–ANN models.

Next, the landslide susceptibility map of the study area was generated by transferring the outputs (i.e., the predicted values of landslide susceptibility index) of the ANN and HHO–ANN to the GIS environment. As is known, there are various techniques for classifying the constructed maps, such as natural break, equal interval, and quantile method. Out of those, equal interval is not practical as it emphasizes one susceptibility class relative to others [50]. Also, the disadvantage of the quantile method lies in placing widely differing values into the same class [51]. Regarding natural break, it reduces the variance within classes and maximizes the variance between classes [52,53], and is the most commonly used method for this aim [54,55,56,57,58]. Hence, using the “natural break” technique, the created maps were classified into five susceptibility categories, namely “Very low”, “Low”, “Moderate”, “High”, and “Very high”. These maps are presented in Figure 7 along with the corresponding histograms. The resulted maps show a good approximation of the location of the marked landslide events. Also, it can be seen that appreciable parts of the area in the West–North and South are found to be under the high risk of landslide.

### 5.3. Performance Assessment of the Models 

**Figure 8:**
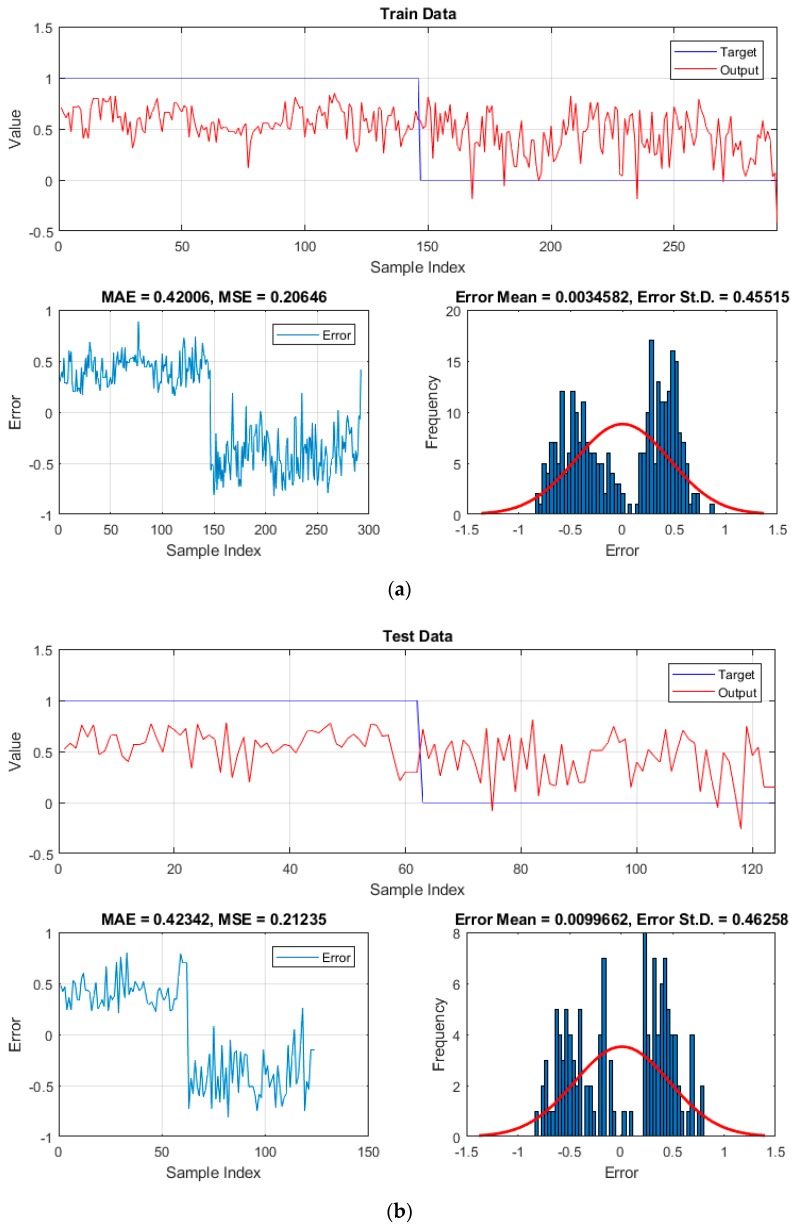

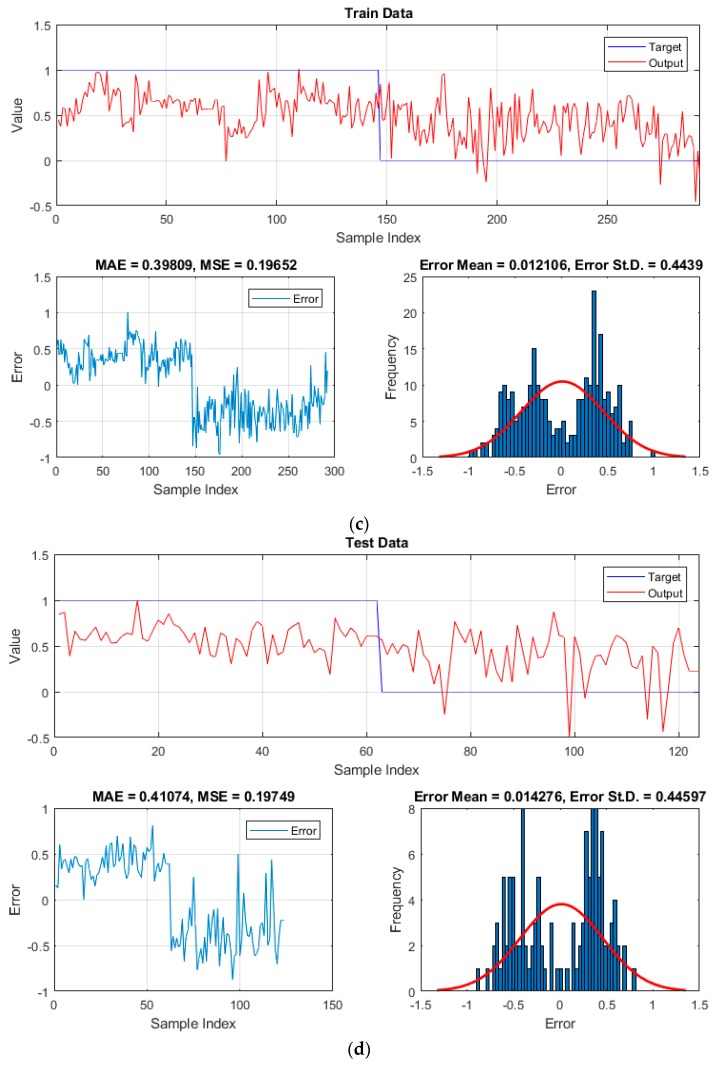
The results obtained for the (**a**,**b**) ANN and (**c**,**d**) HHO–ANN for the training and testing samples, respectively.

Firstly, to measure the performance error of the used models, MSE and MAE error criteria were employed. These values were calculated for both training and testing samples. In this regard, Figure 8 illustrates the comparison between the actual and predicted landslide susceptibility values, as well as the histogram of the errors (i.e., the difference between the mentioned parameters). As is seen, applying the HHO algorithm helped the ANN to have more accurate pattern recognition. Accordingly, the training MSE declined from 0.20646 for the ANN to 0.19652 for the HHO–ANN. The decrease in the calculated MAE (from 0.42006 to 0.39809) provides additional evidence for this claim. As for the testing phase, the decrease of the MSE from 0.21235 to 0.19749 indicates the improvement of the generalization power of the ANN, which results in a more reliable approximation of the unseen landslides. The obtained MAE also attests this statement. 

**Figure 9:**
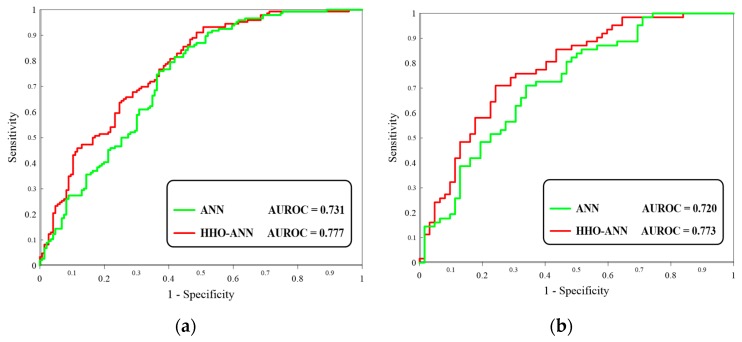
The ROC curves plotted for the (**a**) training and (**b**) testing datasets.

The accuracy of the developed susceptibility maps was also evaluated by drawing the receiver operating characteristic (ROC) curve of both training and testing predictions. The ROC curve plots the specificity (i.e., the proportion of the non-landslide grid cells which are correctly labeled “stable”) versus the sensitivity (i.e., the proportion of the landslide grid cells which are correctly labeled “unstable” [59,60,61]. Beguería [61] stated that the area under the plotted ROC curves (AUROC) is a good indicator of the accuracy of natural hazard modeling. It ranges from 0.5 to 1 directly proportional to the prediction accuracy. Figure 9 displays the plotted ROC curves, as well as the computed AUROC for both ANN and HHO–ANN models (Table 2). Based on this figure, both learning and prediction accuracies of the typical ANN increased after incorporation with the HHO. Accordingly, the accuracy of the training samples rose from 73.1% to 77.7%, and the accuracy of predicting unseen landslides increased from 72.0% (SE = 0.046) to 77.3% (SE = 0.027). The pairwise comparison of the ROC curves was also carried out using the method of Hanley and McNeil [62]. The obtained “significance level” and “z statistic” were 0.1978 and 1.288, respectively.

The percentage of the area which is labeled by each one of the susceptibility classes is also calculated. Based on the generated landslide susceptibility maps, both models have found more than one-third of the studied area to be under the high risk of the landslide (i.e., “High” susceptibility category). Also, more than 20% of the area was classified as “Very high” susceptibility. All in all, the ANN and HHO–ANN classified around 57% (i.e., 4474.50 km^2^) and 54% (i.e., 4210.50 km^2^) of the area as regions with “High” and “Very high” susceptibility. More details of this analysis are presented in Table 3.

Moreover, the percentage of training and testing landslides located in each susceptibility class are presented in Table 4. According to this table, a considerable distinction is the percentage of the landslides found within the “Very high” susceptibility class. As is seen, the prediction of the unreinforced ANN indicates that about 27% and 20% of the training and testing landslides, respectively, are located in the mentioned class. Use of the optimized version increased these values to about 39% and 40%. Also, about 71% and 78% of the landslides which used for pattern discerning by the ANN and HHO–ANN were found in the regions with “High” and “Very high” susceptibility in the developed maps. These values were calculated as 68.87% and 75.98% for unseen landslide events.

### 5.4. Presenting the HHO-Based Predictive Formula 

As previously explained, the main contribution of the used hybrid algorithm (i.e., the HHO) to the problem of landslide susceptibility assessment lies in determining the most proper values of the weights assigned to each landside conditioning factor. Therefore, this section aims to present the landslide susceptibility index formula of the ANN which is optimized by the proposed HHO algorithm. The weights and biases of the HHO–ANN model is shown in Table 5.

Landslide susceptibility index _HHO–ANN_ = 0.2517 × Z1 + 0.9157 × Z2 + 0.3330 × Z3 + 0.1148 × Z4 + 0.4546 × Z5 + 0.5011 × Z6 − 0.6282 × Z7 + 0.6683 × Z8 − 0.3576,(13)

where Z1, Z2, …, Z8 are calculated as follows.

It is well-established that generating a susceptibility map of environmental threats is one of the most fundamental prerequisites for dealing with them. Landslides are one of the most disastrous of these threats. In the case of susceptibility zonation of the landslide, scholars have used different predictive and evaluative techniques. Also, due to the existing shortcomings of statistical methods, as well as the regular machine learning tools, they have employed hybrid metaheuristic algorithms to overcome these drawbacks. These algorithms represent search methods which aim to find the best-fitted solution for a mathematically defined problem.

Artificial neural networks have been efficiently used for landslide susceptibility analysis in various areas. As one of the first attempts, Lee et al. [63] determined the relative importance of landslide thematic factors through training an ANN by the backpropagation algorithm. More recently, Can et al. [64] tested four different learning strategies of the ANN, namely quick propagation, batch backpropagation, Levenberg–Marquardt, and conjugate gradient descent (CGD) for producing the landslide susceptibility map of Ovacık region, Tukey. Their findings revealed that the last algorithm outperforms those presented by other colleagues. However, it also emerged as the slowest algorithm. 

Evolutionary algorithms have also shown good incorporation with typical intelligent models to enhance their performance. Different attempts have conducted the optimization of ANNs and ANFIS for modeling various natural phenomena like landslide [65], forest fire [66,67], and groundwater potential [68,69]. As is known, these population-based algorithms mime different social behaviors to search the most appropriate response to a problem. When it comes to machine learning models, the main objective is to overcome existing drawbacks like local minimum and dimension dangers [23], through achieving more suitable values of computational parameters. It also can be considered as the main contribution of such techniques to the proposed problems. In the case of spatial analysis of landslides, as stated in previous research [63,70], the connecting weights of the ANN denote the relative importance of the thematic layers. In this sense, Moayedi et al. [32] found that utilizing the PSO algorithm enhances the capability of the MLP neural network. They also presented the extracted weights that the PSO assigned to each considered landslide conditioning factor.

In the current research, for the first time, the HHO optimization algorithm was applied to the landslide susceptibility problem by incorporation with ANN. The results clearly showed that the HHO performed efficiently in improving the reliability of the proposed spatial analysis. In investigating the optimization path, the convergence curve of the HHO–ANN ensemble showed that an appreciable portion of the objective function reduction occurred in the first attempts. It continued to decrease the error until it remained more and less steady in the last 200 repetitions. 

Referring to the results, the authors believe that the proposed HHO algorithm can be promisingly used for optimizing different intelligent tools for landslide susceptibility mapping. It seems to be a proper model for assist the engineers and authorities in future planning in order to alleviate the damage caused by landslides. However, the authors believe that the proposed ensemble could achieve higher accuracy by applying some measures like sensitivity analysis for better structures or optimizing the conditioning factors. Last but not least, establishing a comparison between the optimization capability of the HHO with other well-known metaheuristic techniques would be a good idea for future studies.

## 6. Conclusions

Recent years have witnessed the large employment of evolutionary science for optimizing the performance of a typical intelligent model due to the existing drawbacks in dealing with highly complex issues. Landslides are disastrous environmental hazards which need nonlinear analysis to be simulated. Hence, in this paper, a novel metaheuristic technique, namely Harris hawks optimization, was synthesized with an artificial neural network to overcome the computational shortcomings of this model in spatial modeling of landslide susceptibility mapping. After providing the required spatial database, the ANN was coupled with the HHO for adjusting the computational parameters. The results revealed that the HHO acts efficiently in reducing the learning error of the ANN, which resulted in more accurate analysis from the spatial relationship between the landslide occurrence and its conditioning factors. Consequently, the landslide susceptibility map produced by the HHO–ANN was more successful than the ANN map in terms of predicting the unseen landslide events. Finally, due to the acceptable accuracy of the generated maps, they can be used for risk management and decision making in the future.

## Figures and Tables

**Table 1:** Description of the lithology units.

Symbol	Description	Age	Age Era	FR
Qft1	High level piedmont fan and valley terrace deposits	Quaternary	Cenozoic	0.32
OMql	Massive to thick-bedded reefal limestone	Oligocene–Miocene	Cenozoic	5.94
pCmt1	Medium grade, regional metamorphic rocks (Amphibolite Facies)	Pre-Cambrian	Proterozoic	0.00
Kav	Andesitic volcanic	Late Cretaceous	Mesozoic	0.00
Kfsh	Dark grey argillaceous shale	Cretaceous	Mesozoic	0.11
K1m	Limestone, argillaceous limestone, tile red sandstone and gypsiferous marl	Early Cretaceous	Mesozoic	0.00
Plms	Marl, shale, sandstone and conglomerate	Pliocene	Cenozoic	0.00
Klsm	Marl, shale, sandy limestone and sandy dolomite	Early Cretaceous	Mesozoic	2.20
Qft2	Low level piedmont fan and valley terrace deposits	Quaternary	Cenozoic	0.45
E2l	Nummulitic limestone	Eocene	Cenozoic	0.00
Klsol	Grey thick-bedded to massive orbitolina limestone	Early Cretaceous	Mesozoic	0.95
K2av	Andesitic volcanic	Late Cretaceous	Mesozoic	0.00
Murm	Light red to brown marl and gypsiferous marl with sandstone intercalations	Miocene	Cenozoic	5.74
Pd	Red sandstone and shale with subordinate sandy limestone (Dorud FM)	Permian	Paleozoic	0.84
Qal	Stream channel, braided channel, and flood plain deposits	Quaternary	Cenozoic	0.00
PAgr	Granite	Paleocene–Eocene	Cenozoic	0.00
TRKurl	Purple and red thin-bedded radiolarian chert with intercalations of neritic and pelagic limestone (Kerman and Neyzar radiolarites)	Triassic–Cretaceous	Mesozoic	0.00
Kussh	Dark grey shale (Sanandaj shale) (Schist and phyllite)	Late Cretaceous	Mesozoic	1.24
Olc,s	Conglomerate and sandstone	Oligocene	Cenozoic	6.83
Ebv	Basaltic volcanic rocks	Middle Eocene	Cenozoic	3.75
Odi-gb	Diorite to gabbro	Oligocene	Cenozoic	0.00
PeEf	Flysch turbidite, sandstone and calcareous mudstone	Paleocene–Eocene	Cenozoic	1.83
Qcf	Clay flat	Quaternary	Cenozoic	0.22
Kupl	Globotruncana limestone	Late Cretaceous	Mesozoic	0.73
K2l1	Hyporite bearing limestone (Senonian)	Late Cretaceous	Mesozoic	0.00
KPef	Thinly bedded sandstone and shale with siltstone, mudstone limestone and conglomerate	Late Cretaceous–Paleocene	Mesozoic–Cenozoic	0.98
TRKubl	Kuhe Bistoon limestone	Triassic–Cretaceous	Mesozoic	0.85
Oat	Andesitic tuff	Oligocene	Cenozoic	0.89
Pel	Medium to thick-bedded limestone	Paleocene–Eocene	Cenozoic	2.30
TRJvm	Meta-volcanics, phyllites, slate and meta- limestone	Triassic–Jurassic	Mesozoic	0.00
JKl	Crystalized limestone and calc-schist	Jurassic–Cretaceous	Mesozoic	0.00
Kbv	Basaltic volcanic	Early Cretaceous	Mesozoic	0.00
Jugr	Upper Jurassic granite including Shir Kuh granite and Shah Kuh granite	Late Jurassic	Mesozoic	10.90
Ogb	Gabbro	Oligocene	Cenozoic	0.34
OMrb	Red beds composed of red conglomerate, sandstone, marl, gypsiferous marl and gypsum	Oligocene–Miocene	Cenozoic	0.71
pd2	Peridotite including harzburgite, dunite, lherzolite, and websterite	Triassic–Cretaceous	Mesozoic	1.02
E1f	Silty shale, sandstone, marl, sandy limestone, limestone and conglomerate	Early Eocene	Cenozoic	0.88
db	Diabase	Late Cretaceous	Mesozoic	0.00
sr	Serpentinite	Triassic–Cretaceous	Mesozoic	0.00
E1l	Nummulitic limestone	Eocene	Cenozoic	0.00

**Table 2:** The statistical analysis of the testing AUROC.

Methods	Area	Std. Error	*p* Value	Youden Index j	Asymptotic 95% Confidence Interval	
					Lower Bound	Upper Bound
ANN	0.720	0.046	<0.0001	0.3710	0.630	0.809
HHO–ANN	0.773	0.027	<0.0001	0.4247	0.720	0.826

**Table 3:** The ratio and area of each susceptibility class.

Susceptibility Class	ANN		HHO–ANN	
	Ratio (%)	Area (km^2^)	Ratio (%)	Area (km^2^)
Very low	5.12	400.15	5.19	405.59
Low	12.36	965.71	15.27	1192.42
Moderate	25.23	1971.09	25.64	2002.93
High	33.42	2610.36	31.96	2496.24
Very high	23.86	1864.13	21.95	1714.26
High and Very high	57.28	4474.50	53.90	4210.50

**Table 4:** The percentage of the training and testing landslides located in each susceptibility classes.

Susceptibility Class	ANN		HHO–ANN	
	Train	Test	Train	Test
Very low	1.32	1.75	1.52	1.24
Low	6.94	8.76	4.66	6.39
Moderate	21.01	20.62	15.59	16.39
High	43.14	48.76	38.89	35.98
Very high	27.59	20.10	39.34	40.00
High and Very high	70.73	68.87	78.23	75.98

**Table 5:** Weights and biases of the HHO–ANN model.

Neurons (i)	Zi = Tansig (W_i1_ × Elevation + W_i2_ × Slope Degree + W_i3_ × Profile Curvature + W_i4_ × Plan Curvature + W_i5_ × Slope Aspect + W_i6_ × SPI + W_i7_ × TWI + W_i8_ × Land Cover + W_i9_ × Rainfall + W_i10_ × Lithology + W_i11_ × Soil Type + W_i12_ × DTT Road + W_i13_ × DTT Fault + W_i14_ × DTT River + b_i_)														
	W_i1_	W_i2_	W_i3_	W_i4_	W_i5_	W_i6_	W_i7_	W_i8_	W_i9_	W_i10_	W_i11_	W_i12_	W_i13_	W_i14_	b_i_
**1**	0.1389	0.8873	−0.3536	−0.5390	0.6093	0.5324	−0.1268	−0.2768	−0.4110	−0.3422	−0.2914	−0.3273	−0.0984	0.6987	−1.8520
**2**	0.1941	−1.1906	−1.0853	0.8015	0.0674	−0.8543	0.4639	−0.3317	−0.5433	0.5304	−1.0774	−0.9195	0.6389	−0.6643	0.4390
**3**	−0.8748	0.1018	0.9202	−0.4856	−1.0795	0.5776	0.9880	0.8675	−1.1525	−0.0134	0.5032	−0.4689	−1.0977	0.0411	0.9416
**4**	0.6930	1.0847	0.3555	−0.0661	0.4444	0.9255	−1.2186	0.1724	0.0116	−1.1188	1.3439	0.6624	0.2156	1.0617	−1.3524
**5**	−0.6105	−0.0710	0.5563	−2.1150	1.2181	0.2868	0.6106	0.0989	−0.0542	0.7688	−0.3673	−0.8785	1.3454	−0.1275	−0.7786
**6**	0.4460	−0.4059	−0.5671	0.3063	−0.2774	0.4887	−0.6989	−0.3011	0.4759	−0.1634	−1.0011	0.3701	−0.1290	−0.8039	0.2600
**7**	0.1713	−0.7522	0.4283	0.079	0.5879	0.4686	0.5622	−0.3228	1.2865	−0.5585	−0.5446	0.5838	1.0550	0.4543	0.5437
**8**	−1.9718	0.6761	−0.5493	−0.1083	0.6430	−0.6932	−0.2789	−0.9709	0.9544	−0.2919	−0.2008	−0.0433	−0.3142	1.6938	−1.3263

DTT: Distance to the.

## Appendix A

More details about four steps of the exploitation phase in the HHO algorithm are presented below:

1. Soft surround: when $r\geq \frac{1}{2}\;and\;\left|E\right|\geq \;\frac{1}{2}$, then we have:

(A1) $$X\left(iter+1\right)=\Delta X\left(iter\right)-E|JX_{rabit}\left(iter\right)-X\left(iter\right)|$$

(A2) $$\Delta X\left(iter\right)=X_{rabit}\left(iter\right)-X(iter)$$

where the outcome ΔX denotes the difference between the prey position vector and the term *J* stands for the prey jump severity.

2. Hard surround: when $r\geq \frac{1}{2}\;and\;\left|E\right|<\;\frac{1}{2}$, then the current position is expressed as follows:

(A3) $$X\left(iter+1\right)=\Delta X\left(iter\right)-E|JX_{rabit}\left(iter\right)-X\left(iter\right)|$$

3. Advanced rapid dives while soft surround: when $r<\frac{1}{2}\;and\;\left|E\right|\geq \;\frac{1}{2}$, the next action of the hawks is determined by the following relation:

(A4) $$Y=X_{rabit}\left(iter\right)-E|JX_{rabit}\left(iter\right)-X\left(iter\right)|$$

In the following, the below equation describes the diving of the hawks:

(A5) Z=Y+S×LF(D)

where $S_{1\times D}$ shows random vector, *D* symbolizes the issue dimension, and *LF* indicates the levy flight. Let μ and ϑ be random values which can vary from 0 to 1, the *LF* and is calculated as:

(A6) $$LF\left(D\right)=0.01\times \frac{\mu \times \sigma }{{\left|\vartheta \right|}^{\frac{1}{\beta }}}\cdot \;\sigma =\left(\frac{\Gamma \left(1+\beta \right)\times \sin\left(\frac{\pi \beta }{2}\right)}{\Gamma \left(\frac{1+\beta }{2}\right)\times \beta \times 2^{\left(\frac{\beta -1}{2}\right)}}\right)\cdot \beta =1.5$$

Consequently, the hawks’ location is updated by the below equation:

(A7) $$X\left(iter+1\right)=\{\begin{array}{c} Y\;if\;F\left(Y\right)<F\left(X\left(iter\right)\right)\\ Z\;if\;F\left(Z\right)<F\left(X\left(iter\right)\right) \end{array}$$

4. Advanced rapid dives while hard surround: when $r<\frac{1}{2}\;and\;\left|E\right|<\;\frac{1}{2}$, then the behavior of the hawks which are considered to be near the rabbit can be expressed as:

(A8) $$X\left(iter+1\right)=\{\begin{array}{c} Y\;if\;F\left(Y\right)<F\left(X\left(iter\right)\right)\\ Z\;if\;F\left(Z\right)<F\left(X\left(iter\right)\right) \end{array}$$

In the above formula, considering $X_{m}\left(iter\right)=\frac{1}{N}\sum_{i=1}^{N}X_{i}\left(iter\right)$, *Y* and *Z* should be calculated as follow [71]:

(A9) $$Y=X_{rabit}\left(iter\right)-E|JX_{rabit}\left(iter\right)-X_{m}\left(iter\right)|$$

(A10) Z=Y+S×LF(D)

## References

[1] Varnes D.J., Radbruch-Hall D.H. (1976). Landslides cause and effect. Bull. Int. Assoc. Eng. Geol.

[2] Cruden D.M. (1991). A simple definition of a landslide. Bull. Eng. Geol. Environ..

[3] Fell R., Corominas J., Bonnard C., Cascini L., Leroi E., Savage W.Z. (2008). Guidelines for landslide susceptibility, hazard and risk zoning for land-use planning. Eng. Geol..

[4] Pourghasemi H.R., Pradhan B., Gokceoglu C. (2012). Application of fuzzy logic and analytical hierarchy process (AHP) to landslide susceptibility mapping at Haraz watershed, Iran. Nat. Hazards.

[5] Shoaei Z., Ghayoumian J. (1998). The largest debris flow in the world, Seimareh Landslide, Western Iran. Environmental Forest Science.

[6] Hong H., Miao Y., Liu J., Zhu A.X. (2019). Exploring the effects of the design and quantity of absence data on the performance of random forest-based landslide susceptibility mapping. Catena.

[7] Ercanoglu M., Gokceoglu C. (2004). Use of fuzzy relations to produce landslide susceptibility map of a landslide prone area (West Black Sea Region, Turkey). Eng. Geol..

[8] Song Y., Gong J., Gao S., Wang D., Cui T., Li Y., Wei B. (2012). Susceptibility assessment of earthquake-induced landslides using Bayesian network: A case study in Beichuan, China. Comput. Geosci..

[9] Pradhan B. (2013). A comparative study on the predictive ability of the decision tree, support vector machine and neuro-fuzzy models in landslide susceptibility mapping using GIS. Comput. Geosci..

[10] Chen W., Chai H., Sun X., Wang Q., Ding X., Hong H. (2016). A GIS-based comparative study of frequency ratio, statistical index and weights-of-evidence models in landslide susceptibility mapping. Arab. J. Geosci..

[11] Nicu I.C. (2018). Application of analytic hierarchy process, frequency ratio, and statistical index to landslide susceptibility: An approach to endangered cultural heritage. Environ. Earth Sci..

[12] Razavizadeh S., Solaimani K., Massironi M., Kavian A. (2017). Mapping landslide susceptibility with frequency ratio, statistical index, and weights of evidence models: a case study in northern Iran. Environ. Earth Sci..

[13] Youssef A.M., Al-Kathery M., Pradhan B. (2015). Landslide susceptibility mapping at Al-Hasher area, Jizan (Saudi Arabia) using GIS-based frequency ratio and index of entropy models. Geosci. J..

[14] Chen W., Pourghasemi H.R., Naghibi S.A. (2018). A comparative study of landslide susceptibility maps produced using support vector machine with different kernel functions and entropy data mining models in China. Bull. Eng. Geol. Environ..

[15] Vahidnia M.H., Alesheikh A.A., Alimohammadi A., Hosseinali F. (2010). A GIS-based neuro-fuzzy procedure for integrating knowledge and data in landslide susceptibility mapping. Comput. Geosci..

[16] Chen W., Yan X., Zhao Z., Hong H., Bui D.T., Pradhan B. (2019). Spatial prediction of landslide susceptibility using data mining-based kernel logistic regression, naive Bayes and RBFNetwork models for the Long County area (China). Bull. Eng. Geol. Environ..

[17] Oh H.J., Pradhan B. (2011). Application of a neuro-fuzzy model to landslide-susceptibility mapping for shallow landslides in a tropical hilly area. Comput. Geosci..

[18] Pradhan B., Lee S., Buchroithner M.F. (2010). A GIS-based back-propagation neural network model and its cross-application and validation for landslide susceptibility analyses. Comput. Environ. Urban Syst..

[19] Tian Y., Xu C., Hong H., Zhou Q., Wang D. (2019). Mapping earthquake-triggered landslide susceptibility by use of artificial neural network (ANN) models: an example of the 2013 Minxian (China) Mw 5.9 event. Geomat. Nat. Hazards Risk.

[20] Yilmaz I. (2009). Landslide susceptibility mapping using frequency ratio, logistic regression, artificial neural networks and their comparison: a case study from Kat landslides (Tokat-Turkey). Comput. Geosci..

[21] Pham B.T., Bui D.T., Pourghasemi H.R., Indra P., Dholakia M. (2017). Landslide susceptibility assesssment in the Uttarakhand area (India) using GIS: a comparison study of prediction capability of naïve bayes, multilayer perceptron neural networks, and functional trees methods. Theor. Appl. Climatol..

[22] Bui D.T., Tuan T.A., Klempe H., Pradhan B., Revhaug I. (2016). Spatial prediction models for shallow landslide hazards: a comparative assessment of the efficacy of support vector machines, artificial neural networks, kernel logistic regression, and logistic model tree. Landslides.

[23] Chen W., Panahi M., Pourghasemi H.R. (2017). Performance evaluation of GIS-based new ensemble data mining techniques of adaptive neuro-fuzzy inference system (ANFIS) with genetic algorithm (GA), differential evolution (DE), and particle swarm optimization (PSO) for landslide spatial modelling. Catena.

[24] Bui D.T., Tuan T.A., Hoang N.D., Thanh N.Q., Nguyen D.B., Van Liem N., Pradhan B. (2017). Spatial prediction of rainfall-induced landslides for the Lao Cai area (Vietnam) using a hybrid intelligent approach of least squares support vector machines inference model and artificial bee colony optimization. Landslides.

[25] Jaafari A., Panahi M., Pham B.T., Shahabi H., Bui D.T., Rezaie F., Lee S. (2019). Meta optimization of an adaptive neuro-fuzzy inference system with grey wolf optimizer and biogeography-based optimization algorithms for spatial prediction of landslide susceptibility. Catena.

[26] Zhang T., Han L., Chen W., Shahabi H. (2018). Hybrid integration approach of entropy with logistic regression and support vector machine for landslide susceptibility modeling. Entropy.

[27] Tien Bui D., Shahabi H., Shirzadi A., Chapi K., Hoang N.D., Pham B., Bui Q.T., Tran C.T., Panahi M., Bin Ahamd B. (2018). A novel integrated approach of relevance vector machine optimized by imperialist competitive algorithm for spatial modeling of shallow landslides. Remote Sens..

[28] Xi W., Li G., Moayedi H., Nguyen H. (2019). A particle-based optimization of artificial neural network for earthquake-induced landslide assessment in Ludian county, China. Geomat. Nat. Hazards Risk.

[29] Gao W., Guirao J.L.G., Basavanagoud B., Wu J. (2018). Partial multi-dividing ontology learning algorithm. Inf. Sci..

[30] Gao W., Wang W., Dimitrov D., Wang Y. (2018). Nano properties analysis via fourth multiplicative ABC indicator calculating. Arab. J. Chem..

[31] Gao W., Wu H., Siddiqui M.K., Baig A.Q. (2018). Study of biological networks using graph theory. Saudi J. Biol. Sci..

[32] Moayedi H., Mehrabi M., Mosallanezhad M., Rashid A.S.A., Pradhan B. (2018). Modification of landslide susceptibility mapping using optimized PSO-ANN technique. Eng. Comput..

[33] Nguyen H., Mehrabi M., Kalantar B., Moayedi H., Abdullahi M.M. (2019). Potential of hybrid evolutionary approaches for assessment of geo-hazard landslide susceptibility mapping. Geomat. Nat. Hazards Risk.

[34] Shirzadi A., Chapi K., Shahabi H., Solaimani K., Kavian A., Ahmad B.B. (2017). Rock fall susceptibility assessment along a mountainous road: an evaluation of bivariate statistic, analytical hierarchy process and frequency ratio. Environ. Earth Sci..

[35] Rahmati O., Samani A.N., Mahdavi M., Pourghasemi H.R., Zeinivand H. (2015). Groundwater potential mapping at Kurdistan region of Iran using analytic hierarchy process and GIS. Arab. J. Geosci..

[36] Pourghasemi H.R., Kerle N. (2016). Random forests and evidential belief function-based landslide susceptibility assessment in Western Mazandaran Province, Iran. Environ. Earth Sci..

[37] Ercanoglu M., Gokceoglu C. (2002). Assessment of landslide susceptibility for a landslide-prone area (north of Yenice, NW Turkey) by fuzzy approach. Environ. Geol..

[38] Talebi A., Uijlenhoet R., Troch P.A. (2007). Soil moisture storage and hillslope stability. Nat. Hazards Earth Syst. Sci..

[39] Vakhshoori V., Pourghasemi H.R. (2018). A novel hybrid bivariate statistical method entitled FROC for landslide susceptibility assessment. Environ. Earth Sci..

[40] Oh H.J., Kim Y.S., Choi J.K., Park E., Lee S. (2011). GIS mapping of regional probabilistic groundwater potential in the area of Pohang City, Korea. J. Hydrol..

[41] McCulloch W.S., Pitts W. (1943). A logical calculus of the ideas immanent in nervous activity. Bull. Math. Biophys..

[42] (2000). ASCE Task Committee Artificial neural networks in hydrology. II: Hydrologic applications. J. Hydrol. Eng..

[43] Moayedi H., Hayati S. (2018). Modelling and optimization of ultimate bearing capacity of strip footing near a slope by soft computing methods. Appl. Soft Comput..

[44] Moayedi H., Huat B.B., Mohammad Ali T.A., Asadi A., Moayedi F., Mokhberi M. (2011). Preventing landslides in times of rainfall: case study and FEM analyses. Disaster Prev. Manage. Int. J..

[45] Moayedi H., Rezaei A. (2019). An artificial neural network approach for under-reamed piles subjected to uplift forces in dry sand. Neural Comput. Appl..

[46] Moayedi H., Hayati S. (2018). Applicability of a CPT-Based Neural Network Solution in Predicting Load-Settlement Responses of Bored Pile. Int. J. Geomech..

[47] Seyedashraf O., Mehrabi M., Akhtari A.A. (2018). Novel approach for dam break flow modeling using computational intelligence. J. Hydrol..

[48] Heidari A.A., Mirjalili S., Faris H., Aljarah I., Mafarja M., Chen H. (2019). Harris Hawks optimization: Algorithm and applications. Future Gener. Comput. Syst..

[49] Moayedi H., Osouli A., Nguyen H., Rashid A.S.A. (2019). A novel Harris hawks’ optimization and k-fold cross-validation predicting slope stability. Eng. Comput..

[50] Ayalew L., Yamagishi H., Ugawa N. (2004). Landslide susceptibility mapping using GIS-based weighted linear combination, the case in Tsugawa area of Agano River, Niigata Prefecture, Japan. Landslides.

[51] Tehrany M.S., Jones S., Shabani F., Martínez-Álvarez F., Bui D.T. (2019). A novel ensemble modeling approach for the spatial prediction of tropical forest fire susceptibility using logitboost machine learning classifier and multi-source geospatial data. Theor. Appl. Climatol..

[52] Liu J., Duan Z. (2018). Quantitative assessment of landslide susceptibility comparing statistical index, index of entropy, and weights of evidence in the Shangnan area, China. Entropy.

[53] Jenks G.F. (1967). The data model concept in statistical mapping. Int. Yearb. Cartogr..

[54] Irigaray C., Fernández T., El Hamdouni R., Chacón J. (2007). Evaluation and validation of landslide-susceptibility maps obtained by a GIS matrix method: examples from the Betic Cordillera (southern Spain). Nat. Hazards.

[55] Pourghasemi H.R., Pradhan B., Gokceoglu C., Moezzi K.D. (2013). A comparative assessment of prediction capabilities of Dempster—Shafer and weights-of-evidence models in landslide susceptibility mapping using GIS. Geomat. Nat. Hazards Risk.

[56] Xu C., Dai F., Xu X., Lee Y.H. (2012). GIS-based support vector machine modeling of earthquake-triggered landslide susceptibility in the Jianjiang River watershed, China. Geomorphology.

[57] Akgun A., Sezer E.A., Nefeslioglu H.A., Gokceoglu C., Pradhan B. (2012). An easy-to-use MATLAB program (MamLand) for the assessment of landslide susceptibility using a Mamdani fuzzy algorithm. Comput. Geosci..

[58] Jaafari A., Najafi A., Pourghasemi H., Rezaeian J., Sattarian A. (2014). GIS-based frequency ratio and index of entropy models for landslide susceptibility assessment in the Caspian forest, northern Iran. Int. J. Environ. Sci. Technol..

[59] Swets J.A. (1988). Measuring the accuracy of diagnostic systems. Science.

[60] Lasko T.A., Bhagwat J.G., Zou K.H., Ohno-Machado L. (2005). The use of receiver operating characteristic curves in biomedical informatics. J. Biomed. Inf..

[61] Beguería S. (2006). Validation and evaluation of predictive models in hazard assessment and risk management. Nat. Hazards.

[62] Hanley J.A., McNeil B.J. (1983). A method of comparing the areas under receiver operating characteristic curves derived from the same cases. Radiology.

[63] Lee S., Ryu J.H., Won J.S., Park H.J. (2004). Determination and application of the weights for landslide susceptibility mapping using an artificial neural network. Eng. Geol..

[64] Can A., Dagdelenler G., Ercanoglu M., Sonmez H. (2019). Landslide susceptibility mapping at Ovacık-Karabük (Turkey) using different artificial neural network models: comparison of training algorithms. Bull. Eng. Geol. Environ..

[65] Chen W., Pourghasemi H.R., Panahi M., Kornejady A., Wang J., Xie X., Cao S. (2017). Spatial prediction of landslide susceptibility using an adaptive neuro-fuzzy inference system combined with frequency ratio, generalized additive model, and support vector machine techniques. Geomorphology.

[66] Bui D.T., Bui Q.T., Nguyen Q.P., Pradhan B., Nampak H., Trinh P.T. (2017). A hybrid artificial intelligence approach using GIS-based neural-fuzzy inference system and particle swarm optimization for forest fire susceptibility modeling at a tropical area. Agric. For. Meteorol..

[67] Bui Q.T. (2019). Metaheuristic algorithms in optimizing neural network: a comparative study for forest fire susceptibility mapping in Dak Nong, Vietnam. Geomat. Nat. Hazards Risk.

[68] Chen W., Tsangaratos P., Ilia I., Duan Z., Chen X. (2019). Groundwater spring potential mapping using population-based evolutionary algorithms and data mining methods. Sci. Total Environ..

[69] Khosravi K., Panahi M., Bui D.T. (2018). Spatial Prediction of Groundwater Spring Potential Mapping Based on Adaptive Neuro-Fuzzy Inference System and Metaheuristic Optimization. Hydrol. Earth Syst. Sci..

[70] Pradhan B., Lee S. (2010). Regional landslide susceptibility analysis using back-propagation neural network model at Cameron Highland, Malaysia. Landslides.

[71] Du P., Wang J., Hao Y., Niu T., Yang W. (2019). A novel hybrid model based on multi-objective Harris hawks optimization algorithm for daily PM2. 5 and PM10 forecasting. arXiv.

